# Water-pipe smoking promotes epithelial–mesenchymal transition and invasion of human breast cancer cells via ERK1/ERK2 pathways

**DOI:** 10.1186/s12935-018-0678-9

**Published:** 2018-11-13

**Authors:** Khaled W. Sadek, Mahmoud Y. Haik, Anas A. Ashour, Tahira Baloch, Tahar Aboulkassim, Amber Yasmeen, Semir Vranic, Asad Zeidan, Ala-Eddin Al Moustafa

**Affiliations:** 1 College of Medicine, Qatar University, Doha, Qatar; 2 Biomedical Research Centre, Qatar University, Doha, Qatar; 3 Segal Cancer Centre, Lady Davis Institute for Medical Research of the Sir Mortimer B. Davis-Jewish General Hospital, Montreal, QC Canada; 4 Oncology Department, McGill University, Montreal, QC Canada; 5 Syrian Research Cancer Centre of the Syrian Society against Cancer, Aleppo, Syria

**Keywords:** Water-pipe smoking, Breast cancer, EMT, Cell invasion, Cell adhesion, Erk1/Erk2 pathways

## Abstract

**Background:**

With the increasing popularity of water-pipe smoking (WPS), it is critical to comprehend how WPS may affect women’s health. The main goal of this study is to identify the potential outcome of WPS on human breast cancer progression.

**Methods:**

Two breast cancer cell lines, MCF7 and BT20, were used in this investigation. We explored the outcome of WPS on cell morphology and cell invasion using inverted microscope and Biocoat Matrigel invasion chambers. On the other hand, Western blot was employed to study the expression patterns of key control genes of cell adhesion and invasion.

**Results:**

Our data reveal that WPS induces epithelial–mesenchymal transition (EMT) of MCF7 and BT20 breast cancer cell lines; thus, WPS enhances cell invasion ability of both cell lines in comparison with their matched controls. More significantly, WPS provokes a down- and up-regulation of E-cadherin and focal adhesion kinase (FAK), respectively, which are important key regulators of cancer progression genes. Finally, our data point out that WPS incites the activation of Erk1/Erk2, which could be behind the stimulation of EMT and invasion as well as the deregulation of E-cadherin and FAK expression.

**Conclusion:**

Our data show, for the first time, that WPS initiates EMT and stimulates cell invasion of breast cancer cells, which could incite metastatic development in breast cancer patients. Thus, we believe that further studies, both in vitro and in vivo, are required to elucidate the pathogenic outcome of WPS on cancer progression of several human carcinomas including breast.

## Background

Tobacco smoking, although easily preventable, is considered a major cause of morbidity and mortality worldwide, accounting for 6 million deaths each year (World Health Organization). Tobacco smoking today has different forms including cigarette, cigar smoking, e-cigarettes as well as water pipe. Indeed, water-pipe smoking (WPS) is the most common tobacco use in the Middle-East region, and its popularity around the globe is rapidly increasing to the extent that WPS has been described as a global epidemic [1]. Water pipe provides a variety of flower-flavored tobacco in addition to various spices and fruits with some regional and cultural differences [2]. Common misconceptions that consider WPS less harmful than cigarettes exist, which have been pointed out in several studies [2, 3]. Nevertheless, earlier investigations reported the obvious harmful effects of WPS on human health, which are comparable, and maybe even worse, than that of cigarette smoking [4–6]. Thus, meta-analysis reports clearly indicate positive associations between WPS and chronic diseases such as lung, esophageal and bladder cancer, respiratory illness, low birth weight and periodontal diseases [7, 8]. On the other hand, passive smoke from WPS can also cause serious risk of respiratory diseases as well as other health disorders and possibly cancers in exposed nonsmokers [9–11].

To date, it has been well established that cigarette smoking can have multiple adverse effects on human health including cardiovascular and lung diseases as well as several types of cancers such as breast [12–15]. For instance, recent investigations have clearly showed that both active and passive smokers have a higher risk of breast cancer development and mortality related to this disease compared to non-smokers [16–18]. On the other hand, it has been pointed out that cigarette smoking enhances cell invasion and metastatic development of different types of cancer by the initiation of epithelial–mesenchymal transition (EMT) [19–21], which is the hallmark of cancer progression [22]. Thus, it is evident that tobacco smoking can play an important role in the development and progression of several human carcinomas including breast. However, the impact of WPS on breast carcinogenesis has not been investigated yet. Therefore, in this study, we explored, for the first time, the outcome of WPS on breast cancer progression. Our data show that WPS can induce EMT and stimulate cell invasion of human breast cancer cells via the deregulation of several key controller genes of cancer invasion and metastasis. Thus, we believe that more, in vitro and in vivo, investigations are necessary to elucidate the outcome of WPS in breast cancer progression.

## Materials and methods

### Smoking machine protocol and WPS preparation

A standard smoking protocol (Aleppo Method) was used as described previously by our group [23]. The water pipe was prepared by padding the head with 10 gr of brand tobacco mixture known as “*Two Apples*”, covering it with aluminum foil and perforating the foil to allow air passage. A charcoal, “*Three Kings*” brand quick-light briquette, was ignited and placed on top of the head at the beginning of the smoking session. Water in the water bowl was changed at the beginning of every smoking session. The condensate (smoking) was collected using regular laboratory filter paper. Filters were dried and weighed before and after collecting smoke and drying. Afterwards, smoked-filters were solved in PBS or RPMI medium (Qiagen, Toronto, ON) with final concertation of 20 mg/ml of smoking particles; then PBS and RPMI solutions were filtered using 0.45 μm (Costar, USA).

### Cell lines

Two human breast cancer cell lines, MCF7 and BT20, were used in our investigation. Cell lines were obtained from the American Type Culture Collection (Rockville, MD, USA) and maintained in a humidified atmosphere of 5% CO_2_ in air at 37 °C. The cells were routinely cultured in RPMI 1640 supplemented with 10% fetal bovine serum (Life Technologies, Inc., ON, Canada), 2 mM l-glutamine, and 100 µg/ml penicillin–streptomycin. Cancer cells were treated with 100 and 200 μg/ml PBS or RPMI solution of WPS; in parallel, control cells were exposed to the same volume of PBS or RPMI serum free.

### Invasion assay

Cell invasion was assayed in 24-well Biocoat Matrigel invasion chambers (8 µm; Becton–Dickinson, ON, Canada) according to the manufacturer’s protocol. Briefly, cells were incubated with 200 μg of WPS solution, and their control (5 × 10^4^) were plated without WPS solution. Both groups were seeded in the top chamber of Biocoat Matrigel wells. The bottom chamber contained RPMI medium with 10% serum. After 24-h incubation non-invasive cells were removed with a cotton swab while cells that migrated through the membrane and stuck to the lower surface of the membrane were fixed with methanol and stained with 0.5% crystal violet. For quantification, cells were counted under a microscope as previously illustrated by our group [24].

### Clonogenic cell assay

Five hundred cells of MCF7 and BT20 were plated in 6-well plates in duplicates. Cells were washed and fresh medium was added in the presence or absence of 200 μg of WPS solution. The experiment was discontinued when the clones reached 50 cells/clone in each well (~ 7 days), then colonies were fixed and stained with 1.5 ml of 6.0% glutaraldehyde and 0.5% crystal violet. Next, colonies were counted using GelCount (Oxford optronix, UK). The colony fraction (CF) of cells was calculated as previously described [25].

### Western blot analysis

MCF7 and BT20 cells were treated with 200 μg/ml of WPS solution in PBS or serum free RPMI for 3 days, as described above. Afterwards, western blot was performed as previously described by our group [26]. Briefly, 30 μg of protein from each sample was used in this assay. Protein samples were then blotted on a nitrocellulose membrane and detected with anti-E-cadherin and FAK monoclonal antibodies (mAbs) (Bio/Can Scientific), and anti-Erk1/Erk2 phosphotyrosine mAb (Upstate Biotechnology, NY, USA).

### Statistical analysis

Data analyses were performed using SPSS 64-bit version 23 (IBM, NY, USA). Normality of data was confirmed using Shapiro–Wilk test and histograms. Data were analyzed using T-test to determine statistical significance. All tests were two-tailed and results were considered statistically significant if *P*-values were less than 0.05.

## Results

**Fig. 1:**
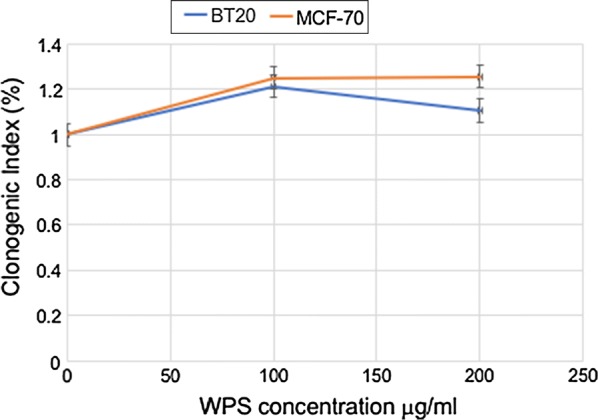
Effects of water-pipe smoking (WPS) on colony formation in human breast cancer cell lines. WPS slightly enhances colony formation of MCF7 and BT20 cell lines in comparison with their control cells. Clonogenic cell assay and WPS exposure were performed as described in “Materials and methods” section

**Fig. 2:**
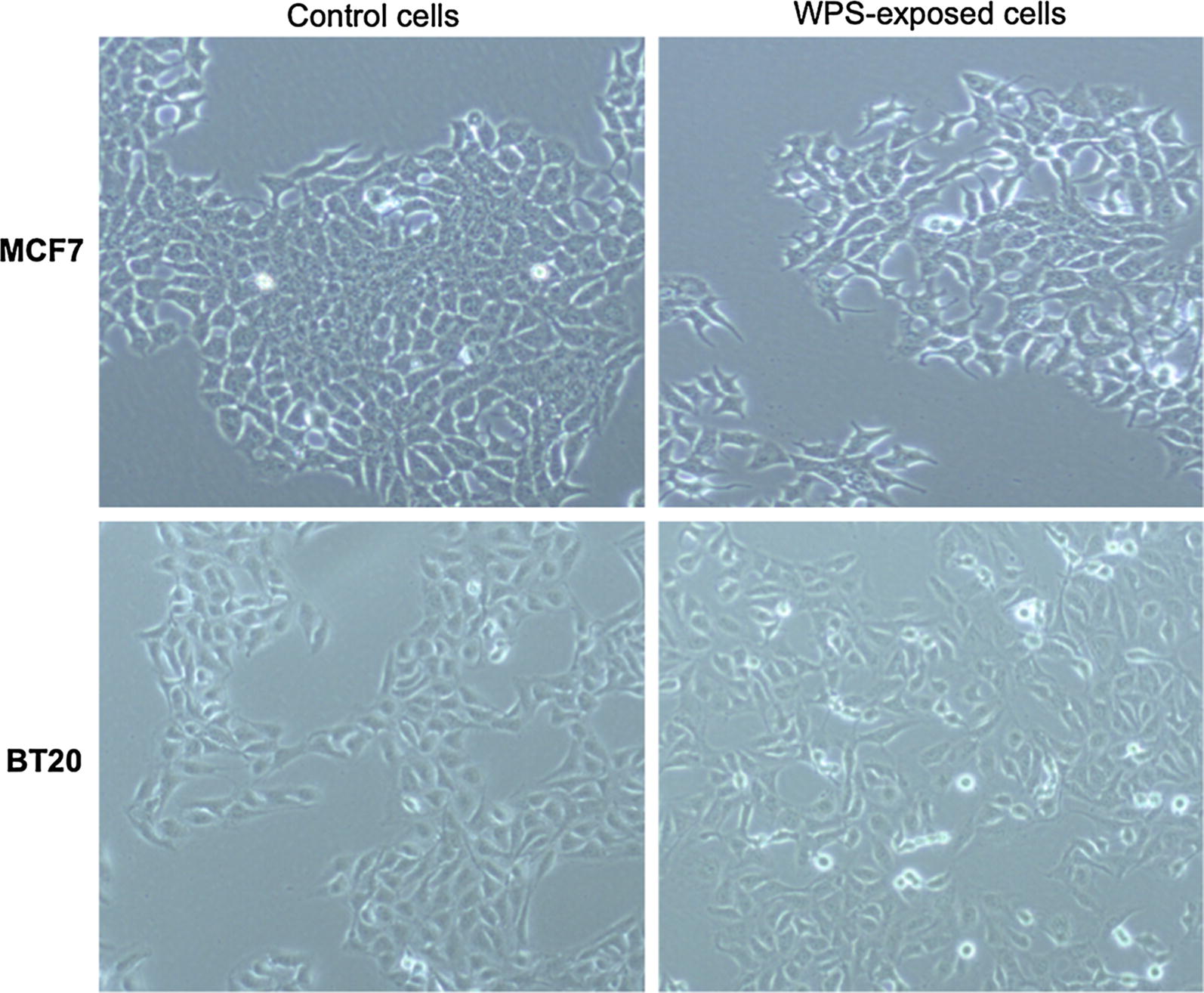
WPS stimulates epithelial–mesenchymal transition (EMT) of breast cancer cell lines, MCF7 and BT20. We note that treatment for 3 days with 200 μg/ml of WPS solution induces morphological changes from “epithelial-like” (control) cells into “fibroblast-like” (mesenchymal) phenotype, which is known as EMT

In order to study the effect of WPS on human breast carcinogenesis, we examined the outcome of WPS on two non- invasive breast cancer cell lines, MCF7 and BT20. Our data revealed that treatment of human breast cancer cells with 100–200 μg/ml of WPS solution for 3 and 8 days slightly deregulates cell cycle progression and increases colony formation, respectively, of MCF7 and BT20 cell lines in comparison with untreated cells (data not shown; Fig. 1). On the other hand, we found that WPS-exposure induces EMT, where both MCF7 and BT20 cells display a more mesenchymal phenotype, and form disorganized multilayered cells in comparison with their matched unexposed controls (Fig. 2). The cells become more elongated in appearance, and show a decrease in cell–cell contact compared with untreated ones.

**Fig. 3:**
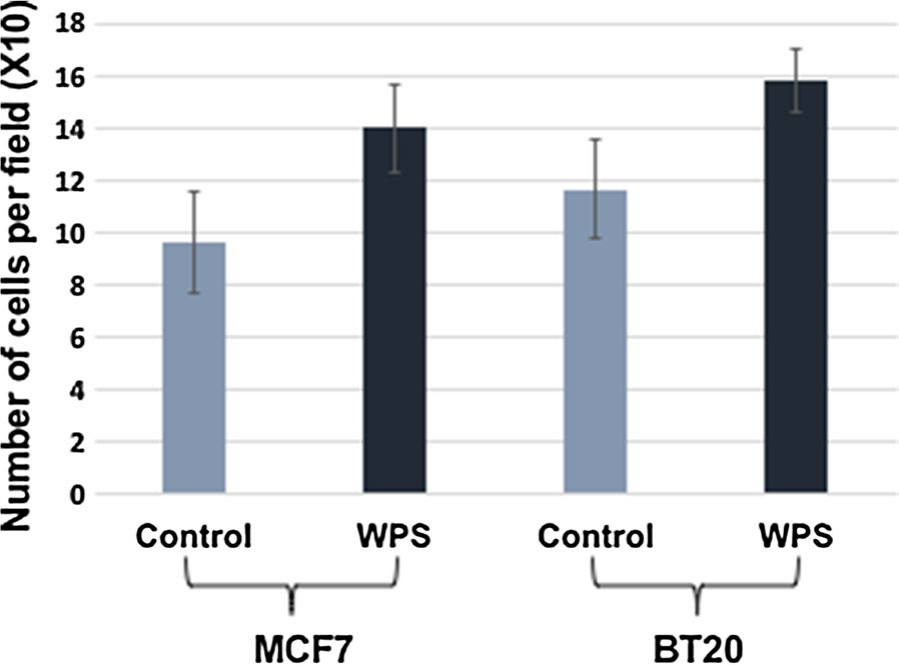
Effect of WPS on cell invasion of human breast cancer cells. The results from matrigel invasion assay indicate that WPS enhances cell invasion ability of MCF7 and BT20 cell lines by approximately 35% in comparison with their control cells. The histograms show mean ± SD (P < 0.002 and 0.001, respectively; t-test was used and is considered significant with P < 0.05). The cancer cells were treated with 200 μg of WPS solution for 2 days as described in “Materials and methods” section

**Fig. 4:**
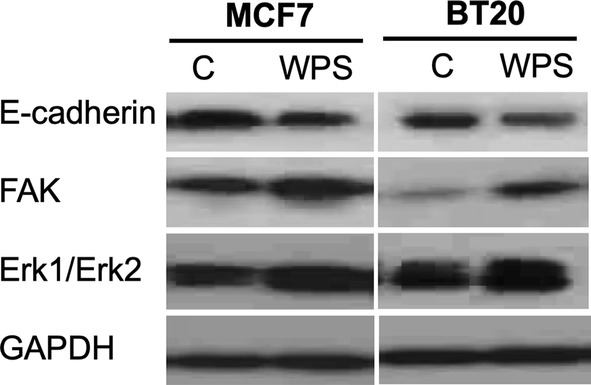
Western blot analysis of E-cadherin, FAK and p-Erk1/Erk2 in MCF7 and BT20 cell lines under the effect of WPS. We notice that WPS decreases/increases the expression patterns of E-cadherin and FAK, respectively, in both cell lines in comparison with control cells; meanwhile, WPS stimulates Erk1/Erk2 phosphorylation in these two cell lines. GAPDH was used as a control. Cells were treated with WPS solution as explained in “Materials and methods” section as well as “Results” section

**Fig. 5:**
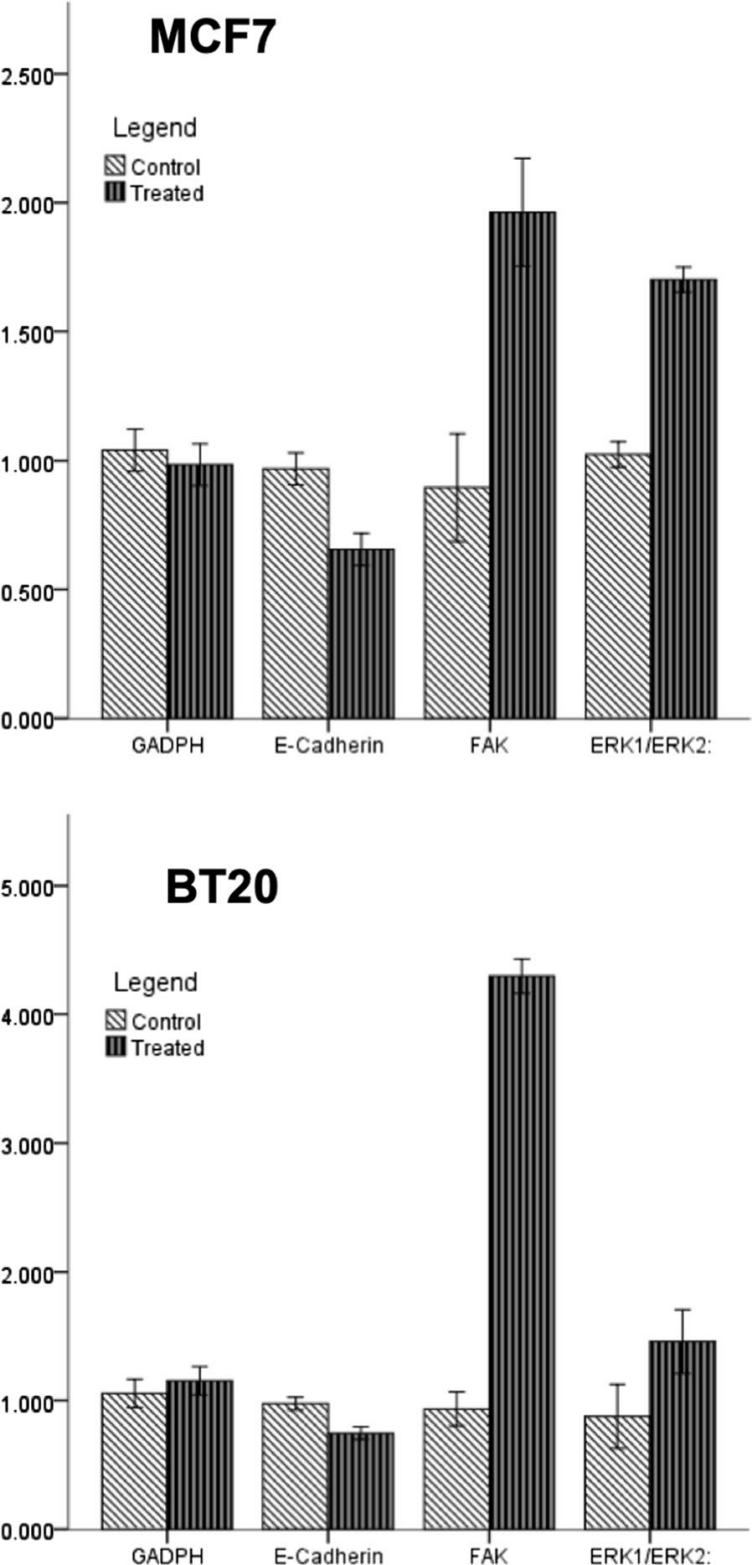
Quantification of the western blot analysis of E-cadherin, FAK and Erk1/Erk2 phosphorylation as well as GAPDH in MCF7 and BT20 exposed to WPS and unexposed (control) cells. This analysis confirms the downregulation of E-cadherin in both cell lines; in parallel, WPS enhances FAK expression and Erk1/Erk2 phosphorylation in the two cell lines in comparison with their control cells. The quantification of both data by ImageJ 64-bit version 1.50b program

To evaluate the role of WPS on cell invasion and migration abilities of human breast cancer cells, matrigel invasion and wound-healing assays were performed. In these experiments, MCF7 and BT20 cells were treated for 48 h with 200 μg/ml of WPS solution. We found that WPS stimulates cell invasion and migration abilities of both cell lines in comparison with their unexposed controls (Fig. 3). Next, we examined the differential expression patterns of E-cadherin and FAK by Western blot analysis of MCF7 and BT20 cells that were either exposed to 200 μg/ml of WPS for approximately 48 h or left unexposed. The results of this analysis were consistently correlated with cell phenotype as well as the invasion and migration ability of both cell lines. Following WPS exposure, the expression of E-cadherin is down-regulated in comparison with unexposed cells, in contrast, FAK expression is up-regulated in MCF7 and BT20 control cells in comparison with WPS-exposed ones, which is consistent with EMT progression (dedifferentiation to mesenchymal phenotype) (Figs. 4, 5). In contrast, unexposed cells show a higher and a lower expression of E-cadherin and FAK, respectively, which is also coherent with EMT progression that enhances cell migration and invasion abilities.

Regarding the mechanisms of WPS on the initiation of EMT and therefore cell invasion in human breast cancer cells, we assumed that the main mechanism behind these events could be Erk1/Erk2 signaling pathways, since it has been reported that tobacco smoking can provoke EMT via Erk1/Erk2 pathways [19, 21]. Thus, Erk1/Erk2 activation was assessed in MCF7 and BT20 cell lines exposed to WPS in comparison with their matched control cells. We found that Erk1/Erk2 is phosphorylated under the effect of WPS, in both cell lines in comparison with their control (Figs. 4, 5). Meanwhile, we noted that there is no significant difference in total Erk1/Erk2 expression between WPS exposed and control cells (data not shown).

## Discussion

In this investigation, we explored for the first time the outcome of WPS on breast carcinogenesis. Indeed, the effect of WPS on breast cancer initiation and/or progression has not been explored yet. Our study revealed that WPS can initiate the EMT event in human breast cancer cells, which is the hallmark of cancer progression and metastasis [22]. Moreover, our data show clearly that WPS stimulate cell invasion of human breast cancer cells. While, numerous recent investigations have demonstrated a strong association between tobacco smoking and breast cancer development and progression in addition to recurrence and mortality [15, 27–29]. Also, it has been revealed that cigarette smoking can enhance EMT of several human carcinoma cells including breast [19, 30–33]. Additionally, earlier studies showed that cigarette smoking could increase breast cancer recurrence by 37%, and therefore increase the overall mortality by 54% compared with nonsmoking patients [16]. Thus, it is evident today that tobacco smoking is an important etiological factor in the development of several types of human cancers inducing lung, oral as well as breast [34]. While, it is important to emphasize that WPS contains the same toxins as cigarette, including high levels of nicotine, heavy metals, particulate matter, and numerous carcinogens, it also adds the adverse effect of charcoal used to heat the tobacco [4–6]. Therefore, WPS can increase health risks by producing high levels of pollutants, such as carbon monoxide, metals and cancer-causing chemicals [35]. Thus, it is evident that WPS can be more detrimental on the development and progression of human cancers as well as cancer-related deaths in comparison with cigarette smoking. Indeed, our study points out that WPS can initiate EMT and therefore enhance cancer invasion ability of two non-invasive breast cancer cell lines. This effect was accompanied with a downregulation of E-cadherin, which is considered an important tumor invasion suppressor. Meanwhile, our data reveal that WPS can enhance the expression pattern of FAK gene; as several investigations reported that FAK is an important key controller gene of cell invasion and metastasis in several types of human carcinomas including breast. Thus, our data concur that cigarette smoking can enhance cancer progression via the initiation of EMT, which is accompanied by the deregulation of E-cadherin, and FAK, as it was clearly demonstrated by several investigations [36–40]. On the other hand, we have recently reported that WPS induces an overexpression of cadherin-6 (CDH6) type 2 gene during embryogenesis [23], which is also involved in the normal development and cancer progression via the initiation of EMT [41–43]. Consequently, we assume that WPS can deregulate the expression pattern of CDH6 type 2 gene in human cancer cells. Taken together, we believe that WPS exposure could have a dramatic effect on the progression of several types of human cancers, including breast, and therefore, cancer mortality.

More significantly, we herein report that WPS activate Erk1/Erk2, which could be the main pathway behind inducing EMT and cell invasion leading to the deregulation of E-cadherin and FAK genes in human breast cancer cells. Herein, it is important to highlight that WPS did not significantly affect the total expression of Erk1/Erk2. Indeed, these data are consistent with previous works regarding cigarette smoking in relation with EMT and Erk1/Erk2 activation, as well as E-cadherin deregulation in various types of human cancer cells including breast [19, 21, 44–46]. Thus, the present study show that Erk1/Erk2 activation is one of the pathways via which WPS can enhance cancer progression and initiate metastasis.

## Conclusions

In conclusion, we herein demonstrate for the first time that WPS can enhance cancer invasion ability of human breast cancer via the initiation of EMT, which is an important event in cancer progression. In parallel, E-cadherin and FAK genes are major targets of WPS in human breast cancer. Finally, our study reveals that this effect can occur via the activation of Erk1/Erk2 pathways. Therefore, it provides evidence that WPS can play a critical role in the progression of human breast cancer. However, further studies are required to elucidate the pathogenic effects of WPS on the development and progression of human carcinomas including breast.
